# Association between red meat consumption and risk of stroke: a meta-analysis of prospective cohort studies

**DOI:** 10.3389/fnut.2026.1797987

**Published:** 2026-06-19

**Authors:** Yi Zhang, Wei Feng, Linxiao Wang, Yiran Zhang, Jing Lv, Shasha Jiang, Heping Zhao, Yan Yu

**Affiliations:** Department of Clinical Laboratory, Honghui Hospital, Xi’an Jiaotong University, Xi’an, China

**Keywords:** ischemic stroke, meta-analysis, processed red meat, prospective cohort study, red meat, stroke

## Abstract

**Introduction:**

Red meat is an important dietary component, with global consumption rising substantially in recent decades. Over the past decades, growing attention has been paid to the potential adverse health effects associated with red meat consumption. Despite numerous epidemiological studies investigating the relationship between red meat consumption and stroke, the impact of red meat consumption on stroke incidence remains inconclusive, and existing meta-analyses synthesizing the evidence for this association are limited. This meta-analysis aimed to summarize the evidence for an association between red meat consumption and stroke risk.

**Methods:**

We searched all prospective studies that provided effect estimates for the potential association between red meat consumption and stroke incidence by conducting a comprehensive search of multiple databases up to Sept 2025, including Web of Science, MEDLINE, EMBASE, and by checking the references of relevant articles. All prospective studies that reported relative risks (RR) and 95% confidence intervals (CI) between red meat consumption and stroke risk were included in our study. A random effects model was used to combine the results.

**Results:**

Overall, 15 studies were included in the meta-analyses, including 1,294,166 subjects and 26,000 stroke cases. Substantial heterogeneity (*I^2^* = 72.5%, *p* < 0.001) was observed across the included studies, and subgroup analyses were performed to explore the potential sources of heterogeneity. We found a modest but significant positive association between red meat consumption and stroke risk (pooled RR = 1.05, 95% CI: 1.00–1.11), robust to sensitivity and publication bias correction. Subgroup analyses showed stronger associations for processed red meat (RR = 1.12), ischemic stroke, and follow-up≥20 years, with heterogeneity by sex and region; no significant associations were observed for unprocessed red meat or hemorrhagic stroke.

**Discussion:**

Our findings support moderate red meat consumption (limiting processed red meat) and provide evidence for stroke prevention dietary guidelines.

**Systematic review registration:**

CRD420261331748.

## 1 Introduction

Stroke is one of the leading causes of death and long-term disability worldwide and remains a major global public health challenge (1). Despite advances in acute treatment and secondary prevention, the burden of stroke continues to increase in many regions, particularly in low- and middle-income countries (2). Because a substantial proportion of stroke burden is attributable to modifiable risk factors, prevention strategies targeting lifestyle and dietary behaviors have attracted increasing attention (3). Among these factors, dietary patterns are especially important because they may influence vascular risk through blood pressure, lipid metabolism, glucose homeostasis, inflammation, and atherosclerotic processes (4, 5).

Red meat is widely consumed globally and represents an important source of high-quality protein and micronutrients, including iron, zinc, and vitamin B12 (6). In general, red meat refers to mammalian meat, including beef, pork, lamb, and veal. However, high consumption of red meat, particularly processed red meat, has been associated with several cardiometabolic diseases (7–9). In this study, unprocessed red meat refers to fresh or minimally processed red meat, whereas processed red meat refers to red meat preserved or modified by smoking, curing, salting, or the addition of chemical preservatives, such as bacon, sausages, hot dogs, luncheon meats, and salami. Total red meat includes both processed and unprocessed red meat (10). Processed red meat may increase exposure to sodium, nitrates, nitrites, and other compounds potentially related to vascular risk (6, 11). Although previous studies and meta-analyses have examined the association between red meat consumption and stroke (12, 13), important uncertainties remain regarding whether the association differs by red meat subtype, including processed and unprocessed red meat, and by stroke subtype, including ischemic and hemorrhagic stroke. In addition, relevant studies also suffer from inconsistent methodologies and flawed study designs, which limit the reliability of their conclusions (12, 14, 15). Therefore, this meta-analysis of prospective cohort studies aimed to systematically evaluate the association between red meat consumption and stroke risk and to explore whether the association varies across red meat subtypes, stroke subtypes, sex, geographic region, and follow-up duration. The results of this study are expected to provide high-quality scientific evidence for the formulation of stroke prevention dietary guidelines and the rational guidance of red meat consumption.

## 2 Materials and methods

### 2.1 Sources and methods of data retrieval

We performed a comprehensive literature search on Web of Science, MEDLINE, EMBASE from the inception dates to Sept 2025. Use the following keywords to identify the included literature assessing the impact of red meat on stroke: red meat, unprocessed red meat, processed red meat, beef, lamb, veal, stroke, ischemic stroke, hemorrhagic stroke, prospective cohort study, and cohort study. The full detailed search strategy for MEDLINE/PubMed is available in Supplementary file 1. Searches for Web of Science and EMBASE were adapted from this core strategy, with targeted adjustments to match the corresponding subject term systems and field retrieval rules of each database, to ensure the consistency and comprehensiveness of literature retrieval.

Exposure categories were classified according to the definitions described in the Introduction. Briefly, total red meat included both processed and unprocessed red meat. When individual studies reported processed and unprocessed red meat separately, these estimates were extracted and analyzed according to the corresponding exposure category.

The entire process of literature retrieval, screening and study selection in this study was strictly conducted in accordance with the PRISMA 2020 statement for systematic reviews and meta-analyses (16). This meta-analysis has been registered in the PROSPERO International Prospective Register of Systematic Reviews (Registration No.: CRD420261331748), and the study design and implementation are fully consistent with the registered protocol.

### 2.2 Inclusion criteria and exclusion criteria

Studies were included if they met the following criteria: (1) clinically diagnosed cases; (2) articles need to be in English; (3) the article was a prospective study; (4) the exposure factor was red meat, including unprocessed red meat and processed red meat; (5) the outcome of was stroke and stroke subtypes; (6) the article reported RR with 95% confidence intervals (CI). Articles meeting the following four criteria will be excluded: (1) studies that did not provide RR with 95% confidence intervals, animal studies, correspondence, reviews, or no raw data; (2) full text of the studies were not available; (3) studies that used other languages except for English; (4) duplicate publications and different follow-up analyses from the same research cohort. Cohort independence was cross-verified by author, study region, cohort name, recruitment period, and sample size. When more than one report from the same cohort was available, we preferentially included the report with the longest follow-up duration. If follow-up duration was similar or overlapping, we selected the report with the largest number of outcome events or the most complete dataset. If multiple reports were still comparable, the study with the most fully adjusted model and the clearest definition of red meat exposure and stroke outcome was retained. When data is missing or the definition of meat is unclear, we will contact the author to provide the relevant data. If critical data (e.g., RRs and 95% CIs) remained unavailable despite attempts to contact the authors, the relevant study or subgroup was excluded. The full text of all relevant literature was independently reviewed by two investigators to decide on inclusion or exclusion, and disagreements were resolved through discussion and, if necessary, collective consultation by all authors. Cohen’s kappa coefficient was calculated to evaluate inter-rater agreement for study selection, and the result showed a kappa value of 0.92 (Supplementary file 2).

### 2.3 Data extraction and quality assessment

We extracted data after identifying the included literature, including the first author, year of publication, study location, sample size, number of cases, and follow-up time. The type of red meat, the type of stroke, and the RR and 95% CI of red meat and stroke were extracted. The quality assessment was independently assessed and scored by two investigators based on the Newcastle-Ottawa Scale (NOS) (17). The NOS has three dimensions, including selection, comparability, and outcomes (cohort studies), and contains a total of eight items. Each project can get one star or 2 stars as long as the conditions are met, and a study can get up to nine stars. Differences in investigators’ scores on quality assessments were re-solved by consensus. Inter-rater reliability for study inclusion was assessed using Cohen’s kappa coefficient (Supplementary file 2). To further improve the rigor of bias assessment, we additionally conducted a supplementary bias evaluation for all included studies based on the 7 core bias domains of the ROBINS-I scale.

### 2.4 Statistical analysis

All data were analyzed using the statistical software Stata (version 12.0, Stata Corp LLC, College Station, TX, United States). The extracted RR with 95% CI was computed from the adjusted RR to explore the relationship between red meat consumption and stroke risk. RRs and 95% CI were pooled using random effects models and differences in stroke risk between the highest and lowest categories of red meat consumption, as defined in each original study. Across the included studies, exposure categories were not uniform and were based on study-specific consumption classifications, such as tertiles, quartiles, quintiles, or predefined cut-off values. When a single study reported multiple effect estimates from the same cohort with overlapping red meat categories, duplicate data points were excluded to avoid double counting of participants.

Heterogeneity was assessed using the Q test and *I*^2^ statistic. Significant heterogeneity was indicated if *I*^2^ > 50% or *p* < 0.05, in which case a random-effects model was used to pool the results; otherwise, a fixed-effects model was employed. In the Q test, *p* < 0.05 was considered significant for heterogeneity, and *I*^2^ values were used to assess the degree of heterogeneity. *I*^2^ values of 25, 50, and 75% indicated low, medium, and high heterogeneity, respectively (18). In addition, exploratory univariable random-effects meta-regression analyses were performed, where feasible, to assess whether study-level characteristics contributed to between-study heterogeneity. We performed a Leave-one-out meta-analysis as a sensitivity analysis to assess the impact of a particular study on the overall results. In addition, potential publication bias was assessed by Egger’s test, in which sensitivity analyses were used to correct the results and assess the effect of bias on the results. Subgroup analyses were performed according to red meat type, stroke type, subject gender, follow-up time, and region. Stratified analyses were performed by red meat type and stroke subtype to separately explore the potential impacts of sources of heterogeneity across the included studies on the observed association results, including inherent variations in red meat definitions and inconsistent reporting of stroke outcomes.

## 3 Results

### 3.1 Study characteristics

**Figure 1:**
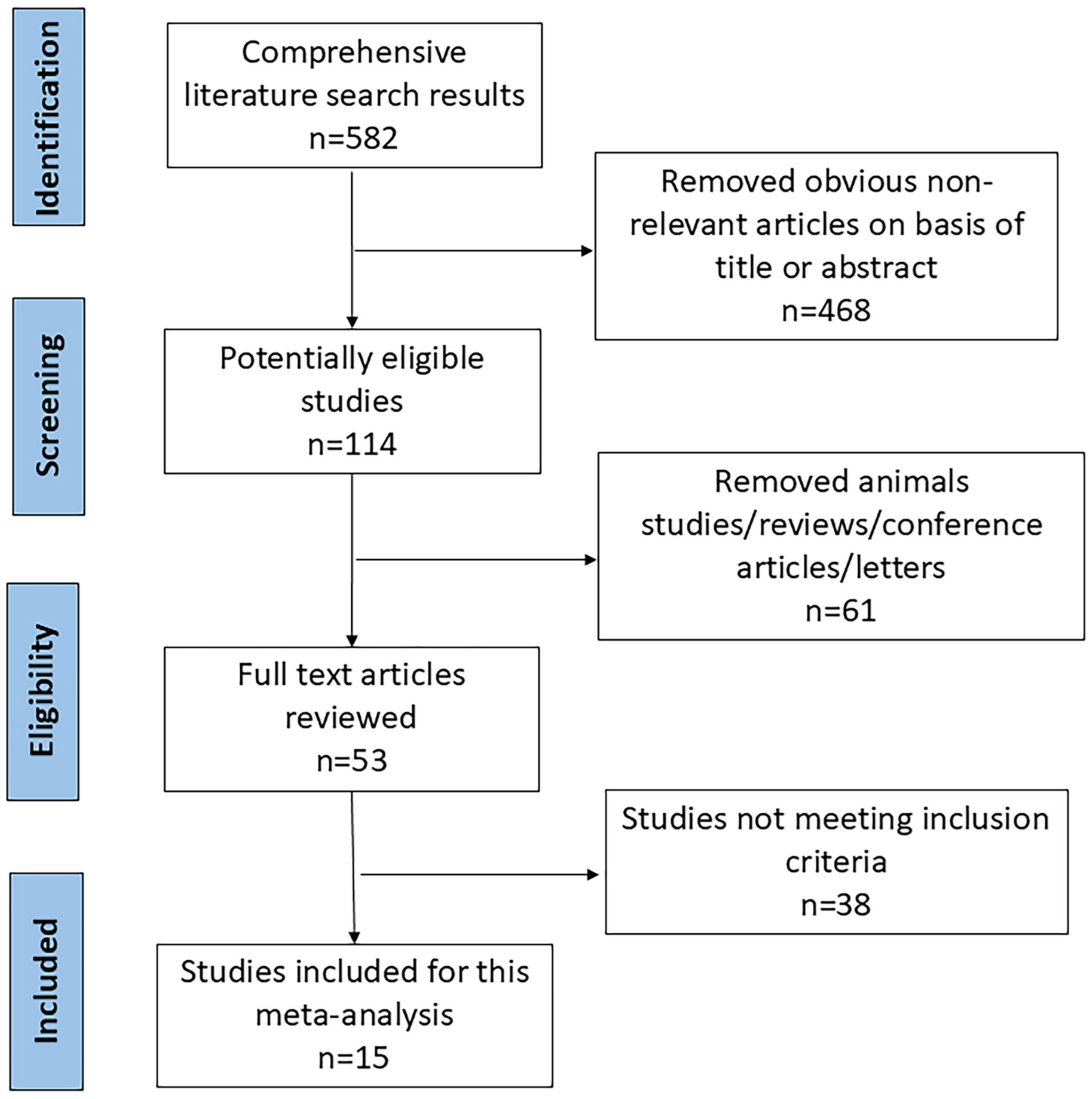
Flow chart of study selection. This flow chart is prepared in accordance with the PRISMA 2020 statement guidelines.

Fifteen prospective cohort studies (19–33) with 1,294,166 subjects and 26,000 cases were included in this meta-analysis. Figure 1 illustrates the process of study selection. Table 1 summarizes the characteristics of all included studies in this meta-analysis. Table 2 presents the NOS scores for the included studies in the quality assessment. In addition, all 15 included prospective cohort studies were subjected to supplementary bias evaluation using the ROBINS-I scale (Supplementary Table S1). The results were consistent with the NOS quality assessment findings, further verifying the high methodological quality of the studies included in this meta-analysis.

**Table 1:** Basic characteristics of the studies included in the meta-analysis.

Author	Year	Nation	Region	Sex	Sample size	Total cases	Follow up year	Outcome	RR (95%CI)	Type of red meat
Wang DD et al.	2024	United States	North America	F/M	148,506	1,261	3.8	Ischemic stroke	1.07 (0.80, 1.43)	Total red meat
							3.8	Ischemic stroke	0.84 (0.59, 1.20)	Unprocessed red meat
							3.8	Ischemic stroke	1.82 (1.25, 2.64)	Processed red meat
Narges Grau et al.	2022	Iran	Asia	F/M	5,432	157	11.25	Total stroke	0.49 (0.31–0.77)	Unprocessed red meat
							11.25	Total stroke	0.52 (0.33–0.82)	Total red meat
Cheng Zheng et al.	2022	United States	North America	F	81,954	2,425	11.3	Total stroke	1.02 (0.94, 1.10)	Unprocessed red meat
							11.3	Total stroke	1.01 (0.93, 1.10)	Total red meat
						1776	11.3	Ischemic stroke	1.03 (0.95, 1.13)	Unprocessed red meat
							11.3	Ischemic stroke	1.02 (0.93, 1.12)	Total red meat
						395	11.3	Hemorrhagic stroke	0.99 (0.82, 1.20)	Unprocessed red meat
							11.3	Hemorrhagic stroke	0.97 (0.79, 1.19)	Total red meat
Sherman J Bigornia et al.	2022	United States	North America	F/M	3,242	83	9.8	Stroke	1.43 (1.07, 1.90)	Unprocessed red meat
Romaina Iqbal et al.	2021	Multinational (21)^^a^	Global (multinational)	F/M	134,297	3,335	9.5	Stroke	1.00 (0.97, 1.02)	Unprocessed red meat
		Multinational (7)^^b^	Global (multinational)	F/M	31,640	475	9.5	Stroke	1.56 (0.94, 2.58)	Processed meat
Priyanka Jain et al.	2020	United States	North America	F	59,727	2,349	26	Total stroke	0.95 (0.91, 0.98)	Unprocessed red meat
							26	Total stroke	0.96 (0.90, 1.02)	Processed red meat
						1,251	26	Ischemic stroke	0.91 (0.86, 0.98)	Unprocessed red meat
							26	Ischemic stroke	0.93 (0.84, 1.02)	Processed red meat
						351	26	Hemorrhagic stroke	1.08 (0.97, 1.16)	Unprocessed red meat
							26	Hemorrhagic stroke	1.02 (0.86, 1.25)	Processed red meat
Tammy Y. N. Tong et al.	2020	Multinational (9)^^c^	Europe	F/M	418,329	4,281	12.7	Ischemic stroke	1.07(0.96, 1.20)	Unprocessed red meat
							12.7	Ischemic stroke	0.95 (0.84, 1.07)	Total red meat
							12.7	Ischemic stroke	0.98 (0.90, 1.07)	Processed red meat
						1,430	12.7	Hemorrhagic stroke	0.95 (0.77, 1.16)	Unprocessed red meat
							12.7	Hemorrhagic stroke	0.95 (0.72, 1.26)	Total red meat
							12.7	Hemorrhagic stroke	1.04 (0.84, 1.29)	Processed red meat
Daniel A Quintana Pacheco et al.	2018	Germany	Europe	F/M	25,540	513	7.5	Stroke	1.09 (0.97, 1.23)	Total red meat
Amiano P. et al.	2016	Spain	Europe	F	25,530	301	13.8	Total stroke	1.21 (0.79, 1.85)	Unprocessed red meat
						229	13.8	Ischemic stroke	1.24 (0.74, 2.05)	Unprocessed red meat
						301	13.8	Total stroke	0.81 (0.51, 1.27)	Processed red meat
						229	13.8	Ischemic stroke	0.82 (0.47, 1.42)	Processed red meat
				M	15,490	373	13.8	Total stroke	0.81 (0.54, 1.21)	Unprocessed red meat
						302	13.8	Ischemic stroke	0.80 (0.51, 1.25)	Unprocessed red meat
						373	13.8	Total stroke	0.92 (0.64, 1.32)	Processed red meat
						302	13.8	Ischemic stroke	0.86 (0.57, 1.29)	Processed red meat
Bernhard Haring et al.	2015	United States	North America	F/M	11,601	699	22.7	Total stroke	1.41(1.04,1.92)	Unprocessed red meat
							22.7	Total stroke	1.24 (0.94, 1.63)	Processed red meat
							22.7	Total stroke	1.38 (1.00, 1.91)	Total red meat
Adam M. Bernstein et al.	2012	United States	North America	F	84,010	2,633	26	Total stroke	1.19 (1.00, 1.41)	Total red meat
						2,633	26	Total stroke	1.10 (0.95, 1.27)	Processed red meat
						2,633	26	Total stroke	1.19 (1.02, 1.40)	Unprocessed red meat
						1,383	26	Ischemic stroke	1.22 (1.06, 1.41)	Total red meat
				M	43,150	1,397	22	Total stroke	1.28 (1.02, 1.61)	Total red meat
						1,397	22	Total stroke	1.27 (1.03, 1.55)	Processed red meat
						1,397	22	Total stroke	1.11 (0.88, 1.39)	Unprocessed red meat
						829	22	Ischemic stroke	1.30 (1.02, 1.64)	Total red meat
				F/M	127,160	4,030	24	Total stroke	1.22 (1.07, 1.40)	Total red meat
						4,030	24	Total stroke	1.15 (1.02, 1.30)	Processed red meat
						4,030	24	Total stroke	1.16 (1.02, 1.33)	Unprocessed red meat
						2,212	24	Ischemic stroke	1.21 (1.07, 1.37)	Processed red meat
Sirin Yaemsiri et al.	2012	United States	North America	F	87,025	1,049	7.6	Ischemic stroke	0.94 (0.87, 1.00)	Total red meat
Susanna C Larsson et al.	2011	Sweden	Europe	M	40,291	2,409	10.1	Total stroke	1.15 (1.00, 1.33)	Total red meat
							10.1	Total stroke	1.23 (1.07, 1.40)	Processed red meat
							10.1	Total stroke	1.07 (0.93, 1.24)	Unprocessed red meat
				M	40,291	350	10.1	Hemorrhagic stroke	1.27 (0.90, 1.80)	Unprocessed red meat
Susanna C Larsson et al.	2010	Sweden	Europe	F	34,670	1,680	10.4	Total stroke	1.12 (0.95, 1.32)	Total red meat
						1,680	10.4	Total stroke	1.07 (0.91, 1.23)	Unprocessed red meat
						1,680	10.4	Total stroke	1.18 (1.00, 1.38)	Processed red meat
						1,310	10.4	Ischemic stroke	1.22 (1.01, 1.46)	Total red meat
						1,310	10.4	Ischemic stroke	1.04 (0.87, 1.23)	Unprocessed red meat
						1,310	10.4	Ischemic stroke	1.24 (1.04, 1.49)	Processed red meat
Ka He et al.	2003	United States	North America	M	43,732	455	14	Ischemic stroke	0.97(0.60,1.55)	Total red meat
				M	43,732	125	14	Hemorrhagic stroke	1.58(0.55,4.55)	Total red meat

CI, confidence interval; F, female; M, male; RR, risk ratio. Data are presented as RR (95% CI). ^^a^Multinational (21): Bangladesh, India, Pakistan, Tanzania, Zimbabwe, Argentina, Brazil, Chile, China, Colombia, Iran, Malaysia, Palestine, Philippines, Poland, South Africa, Turkey, Canada, Saudi Arabia, Sweden, United Arab Emirates. ^^b^Multinational (7): Argentina, Brazil, Canada, Chile, Poland, South Africa, Sweden. ^^c^Multinational (9): Denmark, Germany, Greece, Italy, the Netherlands, Norway, Spain, Sweden, UK.

**Table 2:** Quality assessment of the included studies.

Study	Selection (max 4)	Comparability (max 2)	Outcome (max 3)	Total (max 9)
Wang et al. (19)	4	2	3	9
Grau et al. (20)	4	2	3	9
Zheng et al. (21)	4	2	3	9
Bigornia et al. (22)	4	2	3	9
Iqbal et al. (23)	4	2	3	9
Jain et al. (24)	4	2	2	8
Tong et al. (25)	3	2	3	8
Quintana Pacheco et al. (26)	4	2	3	9
Amiano et al. (27)	4	2	3	9
Haring et al. (28)	4	2	3	9
Bernstein et al. (29)	3	2	3	8
Yaemsiri et al. (30)	4	2	2	8
Larsson et al. (31)	4	2	2	8
Larsson et al. (32)	4	2	2	8
He et al.	4	2	2	8

### 3.2 Meta-analysis

**Figure 2:**
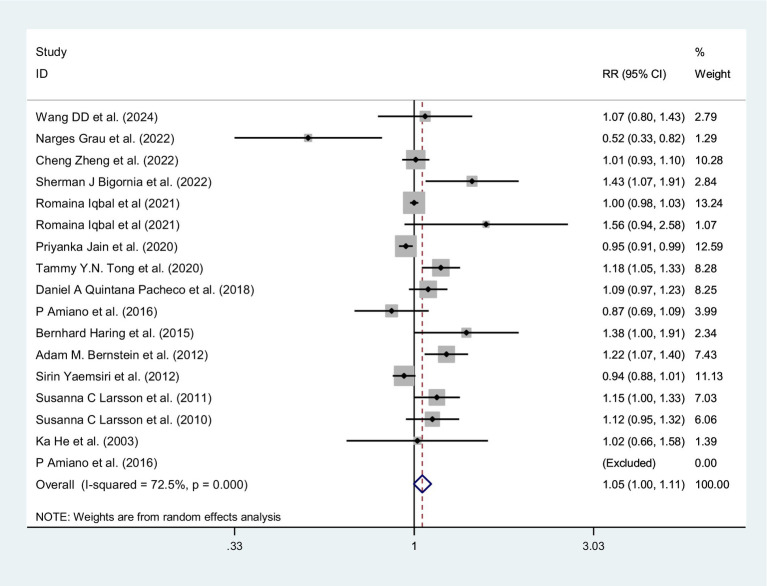
Forest plot describing the association of total red meat with risk for total stroke. RRs of individual studies are indicated by the data markers; Shaded boxes around data markers reflect the statistical weight of the study; 95% CIs are indicated by the error bars; RR with their 95% CI are depicted as a diamond.

The primary random-effects meta-analysis of total red meat consumption and total stroke risk included 17 independent, non-overlapping effect sizes from the 15 eligible prospective cohort studies (some studies reported sex-specific non-overlapping subgroups, which were included independently per our predefined rule) (Supplementary file 3). Significant moderate-to-high heterogeneity was observed, supporting the use of the random-effects model. The pooled relative risk (RR) was 1.05 (95% CI: 1.00–1.11), indicating a statistically significant, slight increase in stroke risk with higher red meat consumption. Individual study effects were generally consistent with the pooled result, and no single study exerted excessive influence on the overall estimate, confirming the robustness of the finding (Figure 2).

### 3.3 Subgroup analysis

**Figure 3:**
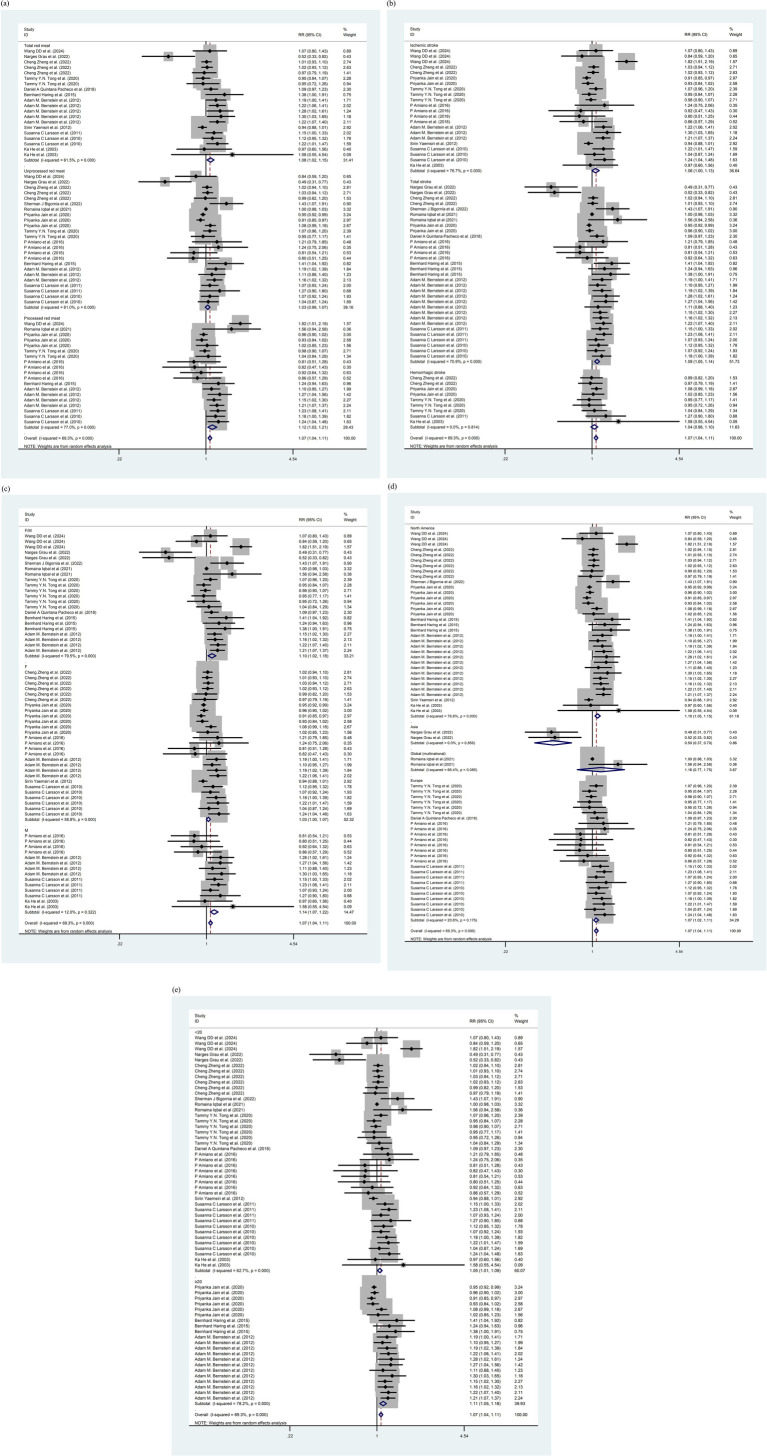
Subgroup analysis stratified by **(a)** type of red meat; **(b)** type of stroke; **(c)** gender; **(d)** area, and **(e)** follow-up duration. RRs of individual studies are indicated by the data markers; Shaded boxes around data markers reflect the statistical weight of the study; 95% CIs are indicated by the error bars; RR with their 95% CI are depicted as a diamond.

Subgroup analyses were performed to investigate heterogeneity sources, including red meat type, stroke subtype, sex, geographic region, and follow-up duration. Processed red meat and total red meat were significantly associated with increased stroke risk, whereas unprocessed red meat showed no significant association (Figure 3a). Ischemic stroke (RR: 1.06, 95% CI: 1.00–1.13) and total stroke were significantly associated with red meat consumption, but hemorrhagic stroke was not (Figure 3b). Subgroup analyses by sex revealed significant associations in female and mixed-sex populations, but not in males (Figure 3c). Geographic differences were observed, with positive associations in North America and Europe, but an inverse association in Asia (likely due to dietary heterogeneity) (Figure 3d). For follow-up duration, studies with follow-up ≥20 years showed a stronger positive association than those with follow-up <20 years (Figure 3e), while the additional analysis using a 10-year cutoff showed consistent positive associations in both the <10-year and ≥10-year subgroups (Supplementary file 4). These findings suggest that the association between red meat consumption and stroke risk varies by red meat type, stroke subtype, population characteristics, and follow-up duration, which partially explains the observed heterogeneity. We further conducted univariable meta-regression analyses, which showed that geographic region and sex were marginally associated with between-study heterogeneity, whereas follow-up duration and publication year were not significant moderators (Supplementary file 5).

### 3.4 Sensitivity analysis

A leave-one-out sensitivity analysis was performed to evaluate the robustness of the pooled result. Briefly, each study was sequentially excluded, and the random-effects meta-analysis was repeated using the remaining studies to recalculate the pooled RR and 95% CI.

After sequential exclusion of each individual study or subgroup, the pooled RR ranged from 1.061 to 1.077, with all 95% CIs overlapping with the original result, indicating no excessive influence of individual studies/subgroups (Supplementary Table S2). Even after excluding the study with the most divergent effect estimate (20), the pooled result remained stable and statistically significant. These findings confirm the robustness of the meta-analytic conclusion.

### 3.5 Publication bias

**Figure 4:**
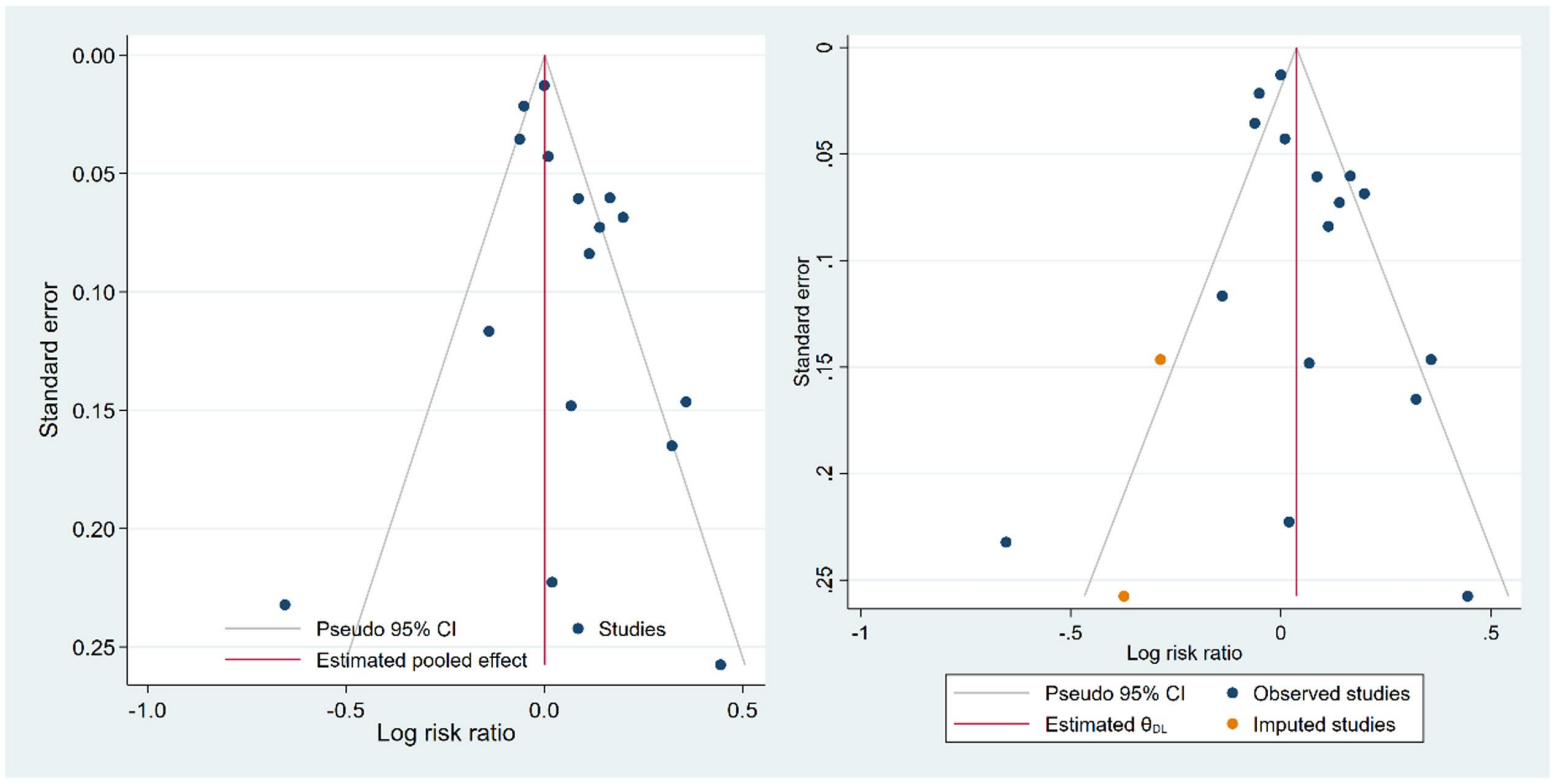
Funnel plots assessing publication bias in the association between red meat consumption and stroke risk. Left: Original funnel plot based on the studies included in the primary meta-analysis. Right: Trim-and-Fill adjusted funnel plot after imputing two potentially missing studies. Blue dots represent observed studies, orange dots represent imputed studies, the grey lines represent pseudo 95% confidence limits, and the red vertical line represents the estimated pooled effect. logRR, logarithm of relative risk; SE, standard error.

Publication bias was assessed based on the studies included in the primary meta-analysis. Visual inspection of the funnel plot showed mild asymmetry (Figure 4). However, Begg’s rank correlation test (*p* = 0.685) and Egger’s regression test (*p* = 0.210) did not indicate significant evidence of publication bias or small-study effects.

The Trim-and-Fill method was further applied as a sensitivity analysis (Figure 4). Two potentially missing studies were imputed on the left side of the funnel plot. After correction, the pooled estimate was attenuated from RR = 1.05 (95% CI: 1.00–1.11) to RR = 1.04 (95% CI: 0.98–1.10) (Supplementary Table S3). These results suggest that potential publication bias did not materially change the direction of the association, although the corrected estimate was weaker and no longer statistically significant.

## 4 Discussion

In this meta-analysis of prospective cohort studies, higher red meat consumption was associated with a modest increase in stroke risk in the primary analysis. The magnitude of the association was small, and the lower confidence limit approached unity; therefore, the finding should be interpreted cautiously. Nevertheless, the direction of the association was generally consistent with previous evidence linking red meat consumption to adverse cardiometabolic outcomes (10, 34, 35). The observed between-study heterogeneity may be partly explained by differences in red meat definitions, stroke outcome classification, population characteristics, dietary patterns, and follow-up duration.

The subgroup analyses provided further insight into potential sources of heterogeneity rather than serving as separate primary conclusions. The association appeared more evident for processed red meat than for unprocessed red meat. This may be partly explained by the higher sodium content and the use of nitrate or nitrite preservatives in processed meat, which have been implicated in hypertension, endothelial dysfunction, oxidative stress, and atherosclerotic processes (36, 37). The significant association with ischemic but not hemorrhagic stroke reflects this may be partly explained by the higher sodium content and the use of nitrate or nitrite preservatives in processed meat, which have been implicated in hypertension, endothelial dysfunction, oxidative stress, and atherosclerotic processes. The distinct pathophysiology of stroke subtypes, as red meat-induced atherosclerosis and hypertension are more strongly linked to ischemic events (13). Sex-specific differences, with significant associations in female and mixed-sex populations but not males, may relate to hormonal influences on lipid metabolism or sex-specific dietary patterns (38, 39). Findings from subgroup analyses stratified by geographic region and follow-up duration should be interpreted with great caution due to the inclusion of single small-sample studies. Therefore, these subgroup findings should be regarded as exploratory and hypothesis-generating, particularly because some subgroup analyses were based on a limited number of studies and may be affected by residual confounding and differences in dietary assessment.

First, while all included primary studies adjusted for major established stroke risk factors, residual confounding from incompletely measured or inconsistently adjusted key factors—including alcohol intake, sodium (salt) consumption, and baseline overall dietary patterns—cannot be fully excluded. These factors are strongly associated with both red meat consumption behaviors and stroke incidence, and heterogeneous adjustment for these confounders across the included studies may introduce residual bias. Critically, the very small magnitude of the observed association makes the effect estimate particularly sensitive to even minor residual confounding, which may contribute to the observed statistical association. Second, the moderate-to-high heterogeneity across studies (*I*^2^ = 72.5%) reflects variability in red meat definitions, stroke subtypes, and population characteristics, which we partially addressed through subgroup analyses. Exploratory meta-regression suggested that geographic region and sex may partly contribute to between-study heterogeneity, whereas follow-up duration and publication year did not show significant effects. However, these findings, which differ from a previous report (40), should be interpreted cautiously given the limited number of independent studies. The findings of publication bias suggest that the observed association may be sensitive to potential funnel plot asymmetry and should be interpreted cautiously, particularly given the small magnitude of the primary pooled estimate. In addition, we compared only the highest versus the lowest categories of red meat consumption, because the exposure units, category definitions, and reported data varied substantially across studies; therefore, a formal dose–response meta-analysis was not feasible in the present study.

Notably, our findings focus on the adverse effects of excessive red meat consumption, while the nutritional value of moderate red meat consumption is well recognized (41–43). Therefore, public health recommendations should emphasize moderation in red meat consumption, particularly limiting processed red meat, to reduce stroke risk while ensuring nutritional adequacy, consistent with existing dietary guidelines (24, 44). One study has also confirmed a declining trend in diet-related ischemic stroke (45). Future studies should prioritize prospective cohorts with standardized red meat assessment, long-term follow-up, and subgroup stratification by stroke subtype and geographic region to refine these associations.

## 5 Conclusion

In conclusion, this meta-analysis confirms a modest but significant positive association between red meat consumption and stroke risk, with stronger effects for processed red meat, longer follow-up, and ischemic stroke. However, the small effect size, substantial heterogeneity, and attenuation after Trim-and-Fill correction indicate that the findings should be interpreted cautiously. Overall, this meta-analysis provides robust evidence that red meat consumption is associated with a small but meaningful increase in stroke risk, with implications for clinical practice and population-level stroke prevention.

## Data Availability

The original contributions presented in the study are included in the article/Supplementary material, further inquiries can be directed to the corresponding authors.
