# Supplementary material for: Association between red meat consumption and risk of stroke: a meta-analysis of prospective cohort studies
Source: Front Nutr. 2026 Jun 19;13:1797987. doi: 10.3389/fnut.2026.1797987 (PMC13327985; doi:10.3389/fnut.2026.1797987)
Supplement: Supplementary file 1 [file Table_1.DOCX]

**Supplementary File 1**

**The search strategy of the present meta-analysis**

The full, detailed search string developed for MEDLINE/PubMed is provided below, which includes standardized MeSH terms, free-text keywords, and boolean operators:

**#1** “Red Meat”[MeSH Terms] OR red meat[Title/Abstract] OR unprocessed red meat[Title/Abstract] OR processed red meat[Title/Abstract] OR beef[Title/Abstract] OR pork[Title/Abstract] OR lamb[Title/Abstract] OR veal[Title/Abstract] OR mutton[Title/Abstract] OR hot dogs[Title/Abstract] OR bacon[Title/Abstract] OR salami[Title/Abstract] OR luncheon meats[Title/Abstract] OR baloney[Title/Abstract]

**#2** “Stroke”[MeSH Terms] OR stroke[Title/Abstract] OR ischemic stroke[Title/Abstract] OR hemorrhagic stroke[Title/Abstract] OR haemorrhagic stroke[Title/Abstract] OR cerebral infarction[Title/Abstract] OR intracerebral hemorrhage[Title/Abstract] OR subarachnoid hemorrhage[Title/Abstract]

**#3** “Prospective Studies”[MeSH Terms] OR prospective cohort study[Title/Abstract] OR cohort study[Title/Abstract] OR follow-up study[Title/Abstract] OR longitudinal study[Title/Abstract]

**#4** #1 AND #2 AND #3

The search strategies for Web of Science and EMBASE were adapted from the above core string, with targeted adjustments to match the corresponding subject term systems (Emtree for EMBASE) and field retrieval rules of each database, to ensure the consistency and comprehensiveness of literature retrieval.
