# Supplementary material for: Association between red meat consumption and risk of stroke: a meta-analysis of prospective cohort studies
Source: Front Nutr. 2026 Jun 19;13:1797987. doi: 10.3389/fnut.2026.1797987 (PMC13327985; doi:10.3389/fnut.2026.1797987)
Supplement: Supplementary file 2 [file Table_2.DOCX]

**Supplementary File 2: Inter-rater Reliability Assessment for Study Selection and Quality Evaluation**

All procedures of study screening, eligibility assessment, and methodological quality evaluation in this meta-analysis were independently performed in duplicate by two investigators with standardized training on the study protocol. Inter-rater reliability was quantified using Cohen’s kappa coefficient (for binary classification of study inclusion/exclusion) and linear weighted Cohen’s kappa coefficient (for ordered categorical quality scores of the Newcastle-Ottawa Scale, NOS). The degree of agreement was interpreted according to the widely recognized Landis-Koch classification standard. All discrepancies between the two investigators were resolved through consensus discussion; unresolved disagreements were adjudicated by collective consultation of all authors.

**1. Inter-rater Reliability for Title/Abstract Screening**

|  | **Investigator 2: Eligible** | **Investigator 2: Excluded** | **Total** |
| --- | --- | --- | --- |
| **Investigator 1: Eligible** | a=106 | b=9 | 115 |
| **Investigator 1: Excluded** | c=8 | d=459 | 467 |
| **Total** | 114 | 468 | N=582 |

The kappa coefficient for title/abstract screening was 0.91 (95%CI: 0.88-0.94), corresponding to almost perfect agreement per the Landis-Koch standard.

**2. Inter-rater Reliability for Full-Text Eligibility Assessment**

Independent assessment results of the two investigators are shown in the 2×2 contingency table below:

|  | **Investigator 2: Included** | **Investigator 2: Excluded** | **Total** |
| --- | --- | --- | --- |
| **Investigator 1: Included** | a=14 | b=1 | 15 |
| **Investigator 1: Excluded** | c=1 | d=37 | 38 |
| **Total** | 15 | 38 | N=53 |

The kappa coefficient for full-text eligibility assessment was 0.92 (95%CI: 0.80-1.04), corresponding to almost perfect agreement per the Landis-Koch standard.

**3. Inter-rater Reliability for Methodological Quality Assessment (NOS Scale)**

Independent NOS scoring results of the two investigators are shown in the table below:

| **Included Study** | **Investigator 1 NOS Score** | **Investigator 2 NOS Score** |
| --- | --- | --- |
| Wang DD et al. (2024) | 9 | 9 |
| Narges Grau et al. (2022) | 9 | 9 |
| Cheng Zheng et al. (2022) | 9 | 9 |
| Sherman J Bigornia et al. (2022) | 9 | 9 |
| Romaina Iqbal et al. (2021) | 9 | 9 |
| Priyanka Jain et al. (2020) | 8 | 8 |
| Tammy Y.N. Tong et al. (2020) | 8 | 8 |
| Daniel A Quintana Pacheco et al. (2018) | 9 | 9 |
| Amiano P et al. (2016) | 9 | 9 |
| Bernhard Haring et al. (2015) | 9 | 9 |
| Adam M. Bernstein et al. (2012) | 8 | 9 |
| Sirin Yaemsiri et al. (2012) | 8 | 8 |
| Larsson et al. (2011) | 8 | 8 |
| Larsson et al. (2010) | 8 | 8 |
| Ka He et al. (2003) | 8 | 8 |

The weighted kappa coefficient for NOS quality assessment was 0.89 (95%CI: 0.82-0.95), corresponding to almost perfect agreement per the Landis-Koch standard.
