# Supplementary material for: Association between red meat consumption and risk of stroke: a meta-analysis of prospective cohort studies
Source: Front Nutr. 2026 Jun 19;13:1797987. doi: 10.3389/fnut.2026.1797987 (PMC13327985; doi:10.3389/fnut.2026.1797987)
Supplement: Supplementary file 3 [file Table_3.DOCX]

**Supplementary File 3 Basic Characteristics of the Study Cohorts Included in the Primary Meta-Analysis**

| Author | Year | Nation | Region | Region2 | Sex | Sample siz | Total cases | Follow up year | Follow up year2 | Outcome | RR | LCI | UCI | Type of red meat |
| --- | --- | --- | --- | --- | --- | --- | --- | --- | --- | --- | --- | --- | --- | --- |
| Wang DD et al. | 2024 | United States | North America | 1 | F/M | 148,506 | 1,261 | 3.8 | <20 | Ischemic stroke | 1.07 | 0.8 | 1.43 | Total red meat |
| Narges Grau et al. | 2022 | Iran | Asia | 2 | F/M | 5432 | 157 | 11.25 | <20 | Total stroke | 0.52 | 0.33 | 0.82 | Total red meat |
| Cheng Zheng et al. | 2022 | United States | North America | 1 | F | 81,954 | 2425 | 11.3 | <20 | Total stroke | 1.01 | 0.93 | 1.1 | Total red meat |
| Sherman J Bigornia et al. | 2022 | United States | North America | 1 | F/M | 3242 | 83 | 9.8 | <20 | Total stroke | 1.43 | 1.07 | 1.9 | Unprocessed red meat |
| Romaina Iqbal et al | 2021 | Multinational (21) | Global (multinational) | 3 | F/M | 134,297 | 3335 | 9.5 | <20 | Total stroke | 1 | 0.97 | 1.02 | Unprocessed red meat |
| Romaina Iqbal et al | 2021 | Multinational (7) | Global (multinational) | 3 | F/M | 31,640 | 475 | 9.5 | <20 | Total stroke | 1.56 | 0.94 | 2.58 | Processed meat |
| Priyanka Jain et al. | 2020 | United States | North America | 1 | F | 59727 | 2439 | 26 | ≥20 | Total stroke | 0.95 | 0.91 | 0.99 | Unprocessed red meat |
| Tammy Y.N. Tong et al. | 2020 | Multinational (10) | Europe | 4 | F/M | 418329 | 7378 | 12.7 | <20 | Total stroke | 1.18 | 1.05 | 1.33 | Total red meat |
| Daniel A Quintana Pacheco et al. | 2018 | Germany | Europe | 4 | F/M | 25540 | 513 | 7.5 | <20 | Total stroke | 1.09 | 0.97 | 1.23 | Total red meat |
| P Amiano et al. | 2016 | Spain | Europe | 4 | F | 25530 | 301 | 13.8 | <20 | Total stroke | 0.98 | 0.76 | 0.76 | Total red meat |
| P Amiano et al. | 2016 | Spain | Europe | 4 | M | 15490 | 373 | 13.8 | <20 | Total stroke | 0.87 | 0.69 | 1.09 | Total red meat |
| Bernhard Haring et al. | 2015 | United States | North America | 1 | F/M | 11601 | 699 | 22.7 | ≥20 | Total stroke | 1.38 | 1 | 1.91 | Total red meat |
| Adam M. Bernstein et al. | 2012 | United States | North America | 1 | F/M | 127160 | 4030 | 24 | ≥20 | Total stroke | 1.22 | 1.07 | 1.4 | Total red meat |
| Sirin Yaemsiri et al. | 2012 | United States | North America | 1 | F | 87025 | 1049 | 7.6 | <20 | Ischemic stroke | 0.94 | 0.87 | 1 | Total red meat |
| Susanna C Larsson et al. | 2011 | Sweden | Europe | 4 | M | 40291 | 2409 | 10.1 | <20 | Total stroke | 1.15 | 1 | 1.33 | Total red meat |
| Susanna C Larsson et al. | 2010 | Sweden | Europe | 4 | F | 34670 | 1680 | 10.4 | <20 | Total stroke | 1.12 | 0.95 | 1.32 | Total red meat |
| Ka He et al. | 2003 | United States | North America | 1 | M | 43732 | 725 | 14 | <20 | Ischemic stroke | 1.02 | 0.66 | 1.58 | Total red meat |
