# Supplementary material for: Association between red meat consumption and risk of stroke: a meta-analysis of prospective cohort studies
Source: Front Nutr. 2026 Jun 19;13:1797987. doi: 10.3389/fnut.2026.1797987 (PMC13327985; doi:10.3389/fnut.2026.1797987)
Supplement: Supplementary file 4 [file Table_4.DOCX]

**Supplementary File 4**

**Supplementary figure 1.** Subgroup analysis by follow-up duration (<10 vs ≥10 years).
