# Supplementary material for: Association between red meat consumption and risk of stroke: a meta-analysis of prospective cohort studies
Source: Front Nutr. 2026 Jun 19;13:1797987. doi: 10.3389/fnut.2026.1797987 (PMC13327985; doi:10.3389/fnut.2026.1797987)
Supplement: Supplementary file 5 [file Table_5.DOCX]

**Supplementary File 5 Univariable random-effects meta-regression analyses for potential sources of heterogeneity in the association between red meat intake and total stroke risk**

| **Variables** | **P** | **Regression coefficients (95% CI)** | **Tau^2^** | **I^2^ residual (%)** |
| --- | --- | --- | --- | --- |
| **Region: Europe** | 0.006 | 0.72 (0.21, 1.23) | 0.011 | 39.29 |
| **Region: Global (multinational)** | 0.009 | 0.71 (0.18, 1.24) | 0.011 | 39.29 |
| **Region: North America** | 0.006 | 0.72 (0.21, 1.22) | 0.011 | 39.29 |
| **Sex: Female** | 0.025 | -0.12 (-0.23, -0.02) | 0.006 | 25.30 |
| **Sex: Male** | 0.417 | -0.07 (-0.24, 0.10) | 0.006 | 25.30 |
| **Follow up year** | 0.823 | 0.00 (-0.01, 0.01) | 0.011 | 39.90 |
| **Publication year** | 0.607 | -0.00 (-0.02, 0.01) | 0.010 | 37.34 |

Note: Data are shown as regression coefficients with 95% confidence intervals. Tau² represents residual between-study variance, and residual I² indicates unexplained heterogeneity. Results should be interpreted cautiously because of the limited number of studies.
